# Choice of Treatment Plan Based on Root Canal Therapy versus Extraction and Implant Placement: A Mini Review

**DOI:** 10.7508/iej.2015.03.001

**Published:** 2015-07-01

**Authors:** Masoud Parirokh, Ahmadreza Zarifian, Jamileh Ghoddusi

**Affiliations:** a *Endodontology Research Center, Dental School, Kerman University of Medical Sciences, Kerman, Iran; *; b *Student Research Committee, Mashhad University of Medical Sciences, Mashhad, Iran; *; c * Dental Research Center, Dental School, Mashhad University of Medical Sciences, Mashhad, Iran*

**Keywords:** Case Selection, Dental Implant, Extraction, Implant, Root Canal Treatment, Tooth Replacement, Treatment Plan

## Abstract

Case selection and treatment plan are important aspects of endodontic treatment. Dentists should organize the treatment plan based on their knowledge, abilities, skills and more importantly the patients’ preference and dentition. Indubitably, the treatment plan for each patient is exclusive and “tailor-made” and cannot be used for all patients. Dentists’ selfestimation of their abilities opens up treatment options; however, in difficult or complicated cases it is advisable to refer to a specialist. Currently, one of the most challenging aspects in dentistry is the choice between extraction and placement of implant (EPI) instead of a complicated root canal treatment (RCT). Overemphasis on one treatment plan while neglecting other options, not only mislead the dentist but also impose unnecessary charges to the patients. This mini-review compares RCT to EPI from various aspects to help practitioners in routine decision making.

## Introduction

Toothache is highly prevalent in the community that makes patients to seek for necessary pain-relieving treatments. Root canal therapy (RCT) and tooth extraction are amongst the most commonly administered treatments for pain relief [1, 2]. During the past two decades new advances such as introduction of biomaterials, application of dental operating microscope (DOM) during surgical and nonsurgical treatments and improvement of engine-driven instruments for root canal preparation have led to higher success rate in endodontic treatment [3-9].

The paradigm shift and increasing tendency of dentists to replace the tooth with implant rather than conventional RCT, has led to a controversy [10]. The increasing number of dentists that think implant may offer better results than RCT has caused a great concern among specialists [11]. 

Up to now, not a single non-biased evidence-based study has been published indicating that extraction and placement of implant (EPI) is more preferential than RCT [12]. Moreover, excessive commercial emphasis on EPI has resulted in an obsessive tendency in dentists to choose it, even for endodontically treatable teeth [11, 13].

In addition, patients’ interest and their ability to afford more expressive treatments may affect their decision-making potential. In a study conducted in Canada, only 39% of patients who had extracted their posterior teeth due to periapical periodontitis, have sought for their replacement with implants [14]. In a recent study in England, most of the patients did not tend to treat their necrotic molars due to high treatment charges and preferred single-tooth edentulism [15]. Based on the above-mentioned studies, it can be concluded that the dentists’ tendency to choose EPI is not always in harmony with the patients’ interest. Therefore, inappropriate guidance from the dentist may result in a toothless patient. Hence, in patients who cannot afford implant, the dentist’s decision should be towards keeping the tooth as long as possible [15]. 

**Figure 1:**
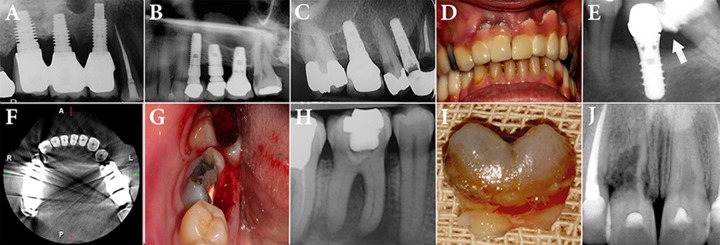
*A)* Inflammation and resorption of the bone surrounding the implant; *B)* Sinus perforation during implantation; *C)* Inappropriate case selection and placement of implant adjacent to a cariously involved root; *D)* Unsatisfactory esthetic following implant placement in anterior portion of maxilla; *E)* Neglecting the inclination of the second mandibular molar and food impaction between the tooth and implant. The dentist tried to expand the composite restoration of the second molar to make a contact between the tooth and the implant, but the patient still suffers from food impaction in that area; *F)* Metallic artifacts due to amalgam in posterior teeth has led to difficulty in observation of adjacent areas: compare the axial view of the anterior teeth to the posterior teeth; *G-I)* Split root and impossibility of restoration makes it very difficult to impossible to treat and place post-core crown for the first mandibular molar; J*)* Severe root resorption in maxillary right incisor makes it impossible to keep the tooth

Incorrect treatment planning may result in implant failure, so the dentists should not always think of implant placement as the ideal treatment. Although implant is a highly successful treatment, failure is probable (Figure 1A-E).

This mini-review tries to compare the RCT and EIP from different aspects and represents some points that every dentist should keep in mind during arranging treatment plan for their patients.

***Post-operative pain and discomfort***

According to the results of a recent study, no significant difference has been observed regarding the presence of post-operative pain and discomfort between RCT and EPI. Patients who underwent RCT reported the maximum pain the day after treatment, while those who underwent EPI reported the maximum pain level by the end of the week after the operation. The quantity of pain in both groups was little and it was due to the difference between the entities of treatment methods [16].

***Duration of treatment***

The time needed for replacement of the tooth with implant is significantly longer than the time required for RCT and placement of permanent restoration; in other words, earlier functional and cosmetic results are expected in RCT, compared to EPI [12]. Despite the decrease in the duration of treatment with the introduction of fresh socket implants, long-term outcomes of this method have not been reported yet [17].

***Mastication force***

Mastication force is significantly stronger in endodontically treated teeth, in comparison with implants [18].

***Cosmetics***

In anterior segment, especially with thin gingival biotype, implant placement is seriously challenged by the cosmetic issues (Figure 1D). In these cases, it seems more appropriate either to keep the tooth and perform RCT or seek for an alternative treatment plan [19].

***Success and survival rate of the treatment***

There is no significant difference regarding the survival rates of RCT and EPI [20]. In a systematic review, the comparison between single tooth implant and endodontic microsurgery showed that during the first 2 to 4 years following the operation, success and survival rate was approximately equal between the two methods; however, in a long-term perspective the success rate of endodontic microsurgery was decreased, while for EPI it had not changed. However, the different criteria of success rate in previous studies obstacles the direct comparison between the two treatments [11, 21].

***Costs***

The treatment costs of EPI are significantly more than RCT and a full coverage permanent restoration [22]. Considering the cost-benefit ratio, RCT and endodontic retreatment are both significantly more appropriate, compared to implant. However, this does not imply to retreatment cases accompanied by periapical surgery [23].

***Quality of life***

No significant difference has been reported regarding the patient’s quality of life between RCT and EPI; patients who underwent either of the treatment methods were content [24].

***The need for complementary treatments***

Endodontically treated teeth have significantly less requirement for complementary treatments after the final restoration, while implant needs more maintenance treatments following the replacement [20].

**Figure 2:**
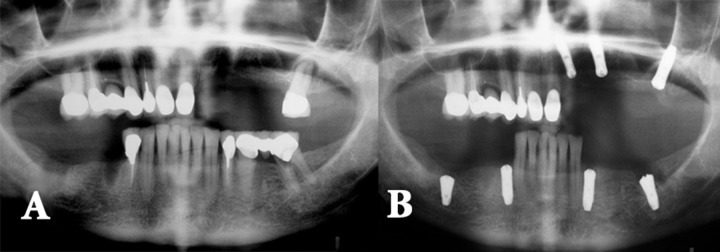
*A)* The dentist did not consider patient’s willingness to keep her teeth; *B)* The dentist replaced many teeth that could be saved with several implants. Patient did not follow the treatment and sued the dentist for not giving her appropriate information prior starting the treatment

***Specific cases***

Patients with high risk of tooth decay or who are vulnerable to periodontal diseases are appropriate cases for EPI. In patients without any systemic, anatomical or economical limitations, implant is recommended instead of RCT [19].

***Predicting factors***

Loosening of the connections between periodontal fibers and gingival disorders are the major prognostic factors of treatment failure in endodontically treated teeth [25]. Besides, patients who have received intravenous bisphosphonate for more than 2-3 years are not suitable candidates for EPI, due to high risk of osteonecrosis. In a recent systematic review, smoking and not returning for periodontal treatment recalls, were the factors that negatively affect the long-term outcomes of EPI [26].

***Patient’s tendency***

In a study on patients with apical periodontitis, most of the patients tended to keep their teeth with RCT and fixed restoration [14]. Therefore, patient’s tendency should be considered even in high risk cases (Figure 2) [27].

The results of a recent retrospective cohort investigation on more than 4000 patients of a dental school, showed that patients’ age, gender and socioeconomic level have significant influences on their choice to receive implant. Patients with high level of socioeconomic status were significantly more likely to receive implant compared to the patients with low level of socioeconomic status. Males and older patients were more interested in EPI compared to the young individuals. The race of the patients also had significant impact on receiving implant compared to the RCT. Caucasians chose EPI more significantly compared to the African-Americans [28]. 

***Effect on radiography***

The use of implant and metals increases the chance for observation of metallic artifacts in x-ray images such as CBCT (Figure 1F) and may distort the image of adjacent structures [29, 30].

***Experience of the practitioner***

Investigations have shown that the operating practitioner’s experience is very important in survival rate of the EPI treatment. In hands of an inexperienced dentist, the survival rate drops to 73%, compared to 95.5% survival rate of implants placed by specialists [31, 32]. 

Comparison of RCT and EPI may imply that in most cases it is better to maintain the tooth, rather than to extract and replace it with an implant. However, in some cases, chances of treatment success are very unlikely or the patient should undertake high expenses for keeping the tooth and meanwhile maintaining the tooth is impossible (Figure 1G-J). In these cases, the dentist may prefer to choose EPI instead of keeping the tooth [14, 23]. In addition, other factors such as tooth restorability, periodontal status and crown/root ratio imply a great impact on practitioner’s decision making. 

Therefore, if the dentist considers a tooth restorable, she/he should inform the patient about the available treatment options and leave the decision up to the patient in order to prevent further misunderstandings. A recent recommendation from pioneer endodontists have shown that the patients should take part in treatment plan and the role of dentist is to honestly give recommendations based on the latest evidence-based documents [12].

## Conclusion

Patient’s preference is of fundamental importance. Some patients prefer not to have extractions at all costs while others avoid high-risk treatments and prefer low risk options. It is the dentists responsibility to involve them in treatment planning by explaining the prognosis of keeping the tooth, costs of treatment and other treatment options to the patients from a professional point of view [22]. Researchers are of the opinion that an evidence-based clinical guideline is required to help the dentists decide on whether to keep the tooth or replace it with an implant, which will probably be introduced in the forthcoming years [19].
